# Linkage to TB and HIV care for persons who smoke illicit drugs: a prospective cohort study

**DOI:** 10.5588/ijtldopen.25.0515

**Published:** 2026-02-11

**Authors:** S. Thomson, V. Overbeck, D. Theron, B. Botha, S. Malatesta, T.C. Bouton, N. Niemand Wolhuter, F. Ratangee, J.I. Campbell, N. Cesare, S. Kulkarni Goodwin, C.S. Meade, C.R. Horsburgh, L.F. White, B. Myers, R.M. Warren, T. Carney, K.R. Jacobson

**Affiliations:** 1 Section of Infectious Disease, Boston Medical Center, Boston University Chobanian and Avedisian School of Medicine, Boston, MA, USA;; 2 Brewelskloof Hospital, Worcester, South Africa;; 3 Department of Biostatistics, Boston University School of Public Health, Boston, MA, USA;; 4 South African Medical Research Council Centre for Tuberculosis Research, Division of Molecular Biology and Human Genetics, Faculty of Medicine and Health Sciences, Stellenbosch University, Cape Town, South Africa;; 5 Section of Pediatric Infectious Diseases, Boston Medical Center, Boston, MA, USA;; 6 Biostatistics and Epidemiology Data Analytics Center, Boston University School of Public Health, Boston, MA, USA;; 7 Department of Psychiatry and Behavioral Sciences, Duke University School of Medicine, Durham, NC, USA;; 8 Departments of Epidemiology and Global Health, Boston University School of Public Health, Boston, MA, USA;; 9 Department of Psychiatry and Mental Health, University of Cape Town, Groote Schuur Hospital, Cape Town, South Africa;; 10 Curtin enAble Institute, Faculty of Health Sciences, Curtin University, Perth, WA, Australia;; 11 Department of Psychology, University of Johannesburg, Johannesburg, South Africa.

**Keywords:** tuberculosis, Worcester, South Africa, people who use drugs, PWUD, care cascade

## Abstract

**BACKGROUND:**

Linking people diagnosed with TB and HIV into care is critical. However, many barriers exist to care linkage, especially for people who use drugs (PWUD).

**OBJECTIVES:**

Characterise differences in TB and HIV care linkage among PWUD in a high-TB/HIV-burden setting.

**DESIGN:**

We analysed HIV and TB linkage to care among PWUD who were diagnosed with HIV and/or TB in a prospective study in Worcester, South Africa. We compared care cascades between participants diagnosed with HIV, TB, or both.

**RESULTS:**

Among 750 participants screened in the community for HIV and TB, we diagnosed and referred 81 individuals with newly diagnosed (N = 39) or previously diagnosed but untreated (N = 42) HIV, as well as 64 individuals with newly diagnosed TB; 11 of these individuals had HIV/TB co-infection. Linkage was higher for TB care (78%) than HIV care (57% for previously diagnosed and 42% for newly diagnosed). 56% of participants with TB had a favourable treatment outcome, whereas only 23% of people with HIV were retained on antiretroviral therapy 6 months after referral.

**CONCLUSION:**

While many PWUD successfully linked to TB and HIV care, disparities exist between the two cascades in this setting. Systems improvements are needed to facilitate linkage and retention for high-risk populations.

To achieve WHO End TB and UNAIDS Global AIDS elimination goals, early disease identification is critical, including detection of asymptomatic, subclinical disease.^1^ Community-based active case finding (ACF) can reduce TB and HIV transmission, but its effectiveness depends on successful linkage to care (LTC), treatment initiation, and treatment completion.^2^ Barriers to LTC include stigma, lack of symptoms, limited health education, cost, and prolonged travel.^1,3^ LTC challenges are amplified among people who use drugs (PWUD), an underserved population in many high-TB/HIV-burden countries. Supporting both HIV and TB care engagement for PWUD is critical, particularly in the setting of co-infection because successful management of one infection without simultaneous successful management of the other compromises outcomes for both. South Africa has among the highest TB and HIV prevalence worldwide.^1,4^ Illicit drug use is also common: a survey in the Western Cape Province reported that 10% of individuals ≥15 years old used illicit drugs in the prior 3 months.^5^ Compared to persons who abstain, PWUD who have TB disease are more likely to delay seeking health care, are more likely to have smear positivity, and are less likely to complete treatment.^6,7^ People with HIV (PWH) who use drugs face additional barriers, including delayed care-seeking and reduced rates of antiretroviral therapy (ART) initiation and poorer adherence.^8–10^ Although the WHO recommends systematic TB screening among PWUD and universal test and treat for HIV, little guidance exists on how to reach or link these individuals to care, especially those identified through community outreach and who are asymptomatic. To date, there is limited information about LTC rates or predictors among PWUD in South Africa or other high-TB/HIV-burden settings.

Here, we characterise the TB and HIV care cascades among PWUD identified via ACF and referred to local clinics for treatment.^11^ We examine factors associated with LTC and delays in treatment access and initiation following TB and/or HIV diagnosis through ACF. Our objective was to describe and compare the care cascades for TB and HIV among PWUD and identify barriers to LTC in each cascade. We explored factors associated with retention to reveal opportunities to strengthen retention in each cascade.

## METHODS

Our study was nested within the Transmission of Tuberculosis Among Drug Use Linkages (TOTAL) study, which enrolled participants in Worcester, South Africa, between April 2021 and October 2023. Detailed methods have been previously published.^11^ In brief, participants in TOTAL were ≥15 years old and self-reported methamphetamine and/or methaqualone use, which was confirmed with urine drug screening. Participants were recruited through respondent-driven sampling through their drug use social network and completed interviewer-administered biobehavioural questionnaires, including the Alcohol, Smoking and Other Substance Involvement Screening Tool (ASSIST), the Center for Epidemiologic Studies Depression Scale (CES-D), and the Household Hunger Scale. We used the WHO four-symptom screening tool to evaluate clinical versus subclinical TB. Prior TB episodes and HIV history were extracted from medical records.

All participants with TB were diagnosed microbiologically on the basis of PCR and/or culture. Participants provided two sputa for Xpert Ultra (Cepheid, Sunnyvale, CA) and mycobacterial culture on separate days. We recorded time-to-culture positivity (TTP) for uncontaminated, culture-positive samples. Blood was drawn for rapid HIV testing (Alere Determine HIV-1/2, Abbott Diagnostics, USA, and HOMEMED HIV 1/2, Homemed Ltd., South Africa), and chest radiographs were performed. Certified study staff provided pre- and post-test HIV counselling.

Referral procedures for both HIV and TB followed study-specific procedures; all research staff were trained on these procedures. For both HIV and TB, participants were referred to their preferred local community health clinics. Study staff referred participants newly diagnosed with HIV, or who were previously diagnosed but not on ART at study enrolment, to local health clinics with appointment booking instructions. Study staff referred participants with untreated TB disease, contacted local clinics for appointments, and offered transportation. Clinics were typically located in the neighbourhoods in which participants lived. While the processes for LTC for HIV and TB differed, these processes mirrored programmatic conditions in our setting.

We characterised four steps of the HIV care cascade: 1) referral to care (all participants with new or untreated HIV, per study protocol), 2) LTC, defined as accessing HIV care within 6 months of referral, 3) ART initiation or re-initiation, and 4) retention on ART. We defined loss to follow-up for PWH as the absence of treatment, medication refill, or follow-up visit for ≥3 months. We defined three steps of the TB care cascade: 1) referral to care (all participants with a diagnosis of TB, per study protocol), 2) LTC, defined as accessing TB care within 6 months of referral, and 3) favourable TB treatment outcome, defined as disease cure or treatment completion. In this study, we include only participants who were referred for TB and/or HIV treatment and who were enrolled for at least 6 months in the TOTAL study to allow sufficient time for outcomes to be assessed. Treatment initiation and outcomes were abstracted from medical records.

We generated independent care cascades for HIV and TB. We further stratified the HIV care cascade by whether participants were previously diagnosed with HIV but not receiving ART versus newly diagnosed with HIV. We used descriptive statistics to analyse linkage completion and timing and to examine associations between participant demographic characteristics and LTC.

### Ethical statement

Stellenbosch University, Boston University/Boston Medical Center, the South African Medical Research Council, University of Cape Town, and the Western Cape Department of Health granted ethical approval.

## RESULTS

Among 750 participants enrolled in the TOTAL study, we diagnosed and referred for care 81 individuals with HIV and 64 with TB, including 11 participants with TB/HIV co-infection (Supplementary Data Figure S1). Of 81 participants referred for HIV care, 39 (48%) were newly diagnosed, while 42 (52%) had been previously diagnosed but were not receiving ART. An additional 49 PWH were receiving ART at enrolment and so were not referred.

The completion of the HIV and TB care cascades is illustrated in Figure. A greater proportion of participants referred for TB care successfully linked (N = 50, 78%) compared to those referred for HIV care (N = 41, 51%). Of 39 newly diagnosed PWH, 17 (44%) linked to care. Of these participants, 15 (88%) started ART, of whom 8 (53%) were retained in care for at least 6 months. Of 42 participants with previously diagnosed HIV not receiving ART, 24 (57%) linked to care. Of these participants, 22 (92%) restarted ART, of whom 9 (41%) were retained in care at 6 months. Of 64 participants with TB, 50 (78%) successfully linked to care, of whom 36 (72%) had known favorable outcome. PWH who were diagnosed with culture-positive TB were significantly more likely to link to HIV care (*P* = 0.006) than those diagnosed with HIV only (Supplementary Data Table S1).

**Figure.**
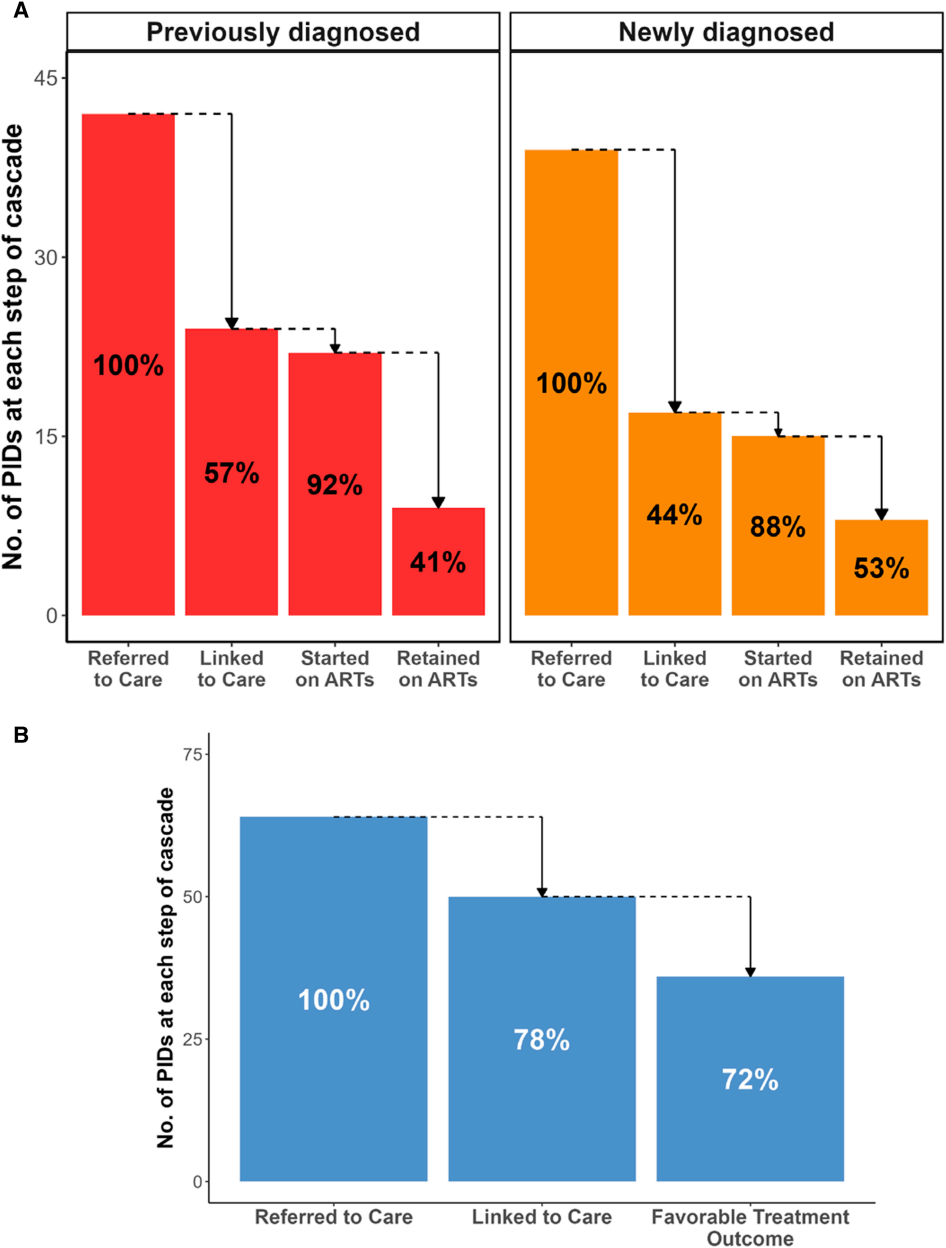
**A:** Care cascade among people who use drugs (PWUD) with HIV. Percents refer to the percentage of participants from the previous cascade step. **B:** Care cascade among PWUD with TB. Percents refer to the percentage of participants from the previous cascade step. ART = antiretroviral therapy; PID = participant.

Supplementary Data Table S1 summarises characteristics of participants who did and did not link to HIV care. Among those referred for HIV care, 79 (98%) reported methamphetamine use and 75 (93%) methaqualone use. All participants reported smoking these drugs. Those who linked to HIV care trended towards having lower alcohol risk scores (*P* = 0.079) and were more likely to be normal rather than underweight (76% vs. 51%, *P* = 0.024). Notable delays occurred between referral and initial HIV evaluation, both among those who were previously diagnosed (median: 220 days [interquartile range (IQR): 122, 412]) and newly diagnosed with HIV (median: 92 days [IQR: 49, 315]) (Table 1). Delays also occurred between referral to treatment initiation (previously diagnosed: 290 days [IQR: 137, 420], N = 20; newly diagnosed: 175 days [IQR: 54, 491], N = 14).

**Table 1.** HIV linkage information among those with a previous versus new HIV diagnosis.

	Previously diagnosed – linked to care (N = 24)	Newly diagnosed – linked to care (N = 17)
Days from referral to initial evaluation	220 (122, 412)	92 (49, 315)
Initiated ARTs	22 (91.7%)	15 (88.2%)
Days from referral to ART initiation (N = 20, N = 14)	290 (137, 420)	175 (54, 491)
Lost to follow-up (N = 22, N = 14)	13 (59.1%)	6 (42.9%)
Nadir CD4 count (N = 21, N = 15)	215 (75, 322)	303 (212, 391)

ART = antiretroviral therapy.

Supplementary Data Table S2 summarises characteristics of participants who did and did not link to TB care. Among those referred to TB care, 58 (91%) reported methamphetamine use and 61 (95%) reported methaqualone use. Of those referred for TB treatment, more than half were asymptomatic (N = 37, 59%). Only 15 (23%) participants were smear positive, and median TTP on culture was 10.0 days (IQR: 7.0, 16.0), reflecting lower disease burden at diagnosis. Participants who linked to TB care were significantly more likely to have moderate/severe alcohol use (28% vs 0%, *P* = 0.025) and were more likely to report coughing (36% vs. 7%, *P* = 0.037) compared to those who did not link (Supplementary Data Table S2). Those who linked to initial TB clinic evaluation (N = 50) did so within a median 2 days (IQR: 1, 10) from referral and quickly initiated treatment (median: 4 days [IQR: 2, 14]; N = 47) (Table 2).

**Table 2.** TB linkage information.

	Overall (N = 50)
Days from referral to initial evaluation (median, IQR)	2 (1, 10)
Sputum collection at clinic	40 (80.0%)
Initiated treatment	48 (96.0%)
Days from referral to treatment initiation (N = 47)	4 (2, 14)
Completed treatment at clinic #1 (N = 48)	33 (68.8%)
Treatment outcome (N = 48)	
Cured/treatment completed	36 (75.0%)
Lost to follow-up	10 (20.8%)
Died	1 (2.1)
Moved/transferred out	1 (2.1%)

IQR = interquartile range.

## DISCUSSION

In this analysis of HIV and TB care linkage among PWUD, we found higher linkage to and retention in TB than to HIV care. We also found markedly longer time to HIV versus TB referral completion and treatment initiation. Although most participants with TB were asymptomatic, three quarters linked to and remained in TB care, demonstrating that PWUD diagnosed in the community in high TB and HIV settings can be successfully engaged in care. Participants with TB/HIV co-infection had significantly higher HIV care linkage than participants living with HIV only. Those reporting TB symptoms were more likely to link to TB care.

Our findings align with existing literature that has shown lower HIV than TB linkage in South Africa, even with integrated services.^11,12^ Increased LTC for PWH with TB disease may stem from co-management by the same provider when TB treatment begins. A recent meta-analysis found that co-located TB and HIV services improve linkage and patient retention for both diseases.^13^ Importantly, the same factors leading to differences in HIV versus TB care linkage may have contributed to differences in treatment outcomes. While our primary outcomes for TB and HIV are not directly comparable, it is notable that among those who initiated treatment, 72% with TB had favourable outcomes, compared to just half of PWH remaining in HIV care 6 months after referral. Notably, our LTC rates for both HIV and TB were similar to LTC rates among the general population in South Africa, which have been estimated to be 46%–60% for HIV^14,15^ and 67%–76% for TB.^16^ Despite these comparable rates, it is likely that PWUD continue to face unique hurdles to accessing both HIV and TB care. Future studies on LTC in this setting should consider gathering and stratifying results by substance use behaviour to further clarify specific barriers in this population.

Our results indicate that co-located services may also benefit those with subclinical TB who use drugs. Engaging and maintaining PWUD in TB/HIV care can be challenging, but service integration, voucher incentives, and peer support improve linkage.^17^ Peer recruitment methods like respondent-driven sampling have successfully engaged PWUD for HIV screening.^18–20^ Our high TB linkage rates suggest that with adequate support and education, PWUD are motivated to seek care. While ACF improves diagnosis, effective LTC requires additional efforts. For instance, a study on community-based TB ACF in South Africa found that diagnostic counselling and referral letters alone were insufficient, emphasising the need for follow-up and incentives.^21^ Researchers in Malawi found that individuals with subclinical TB are motivated to receive care, prioritising effective TB treatment with minimal side effects and transmission risk, elements that should be built into educational components of treatment strategies.^22^ More research is needed to understand how ACF impacts LTC for HIV and TB, especially among PWUD.

We identified factors associated with linkage gaps among PWH and participants with TB. Troublingly, PWH who were underweight were less likely to link to care, mirroring findings from other studies.^23,24^ Pathways underlying this association are likely multifactorial, including mental health and socio-economic factors that inhibit both food security and health care access. Others have studied nutritional support for PWH, though further research is needed to identify effective interventions for PWH facing undernutrition, including those with concomitant substance use.^25–27^ Although there was no significant difference in linkage to TB care between symptomatic and asymptomatic participants, we found that presence of cough was associated with increased LTC. A recent cohort study from South Africa found that people with cough, but not other TB symptoms, had twice the odds of linking to outpatient TB care after inpatient diagnosis compared to people without cough.^28^ Our results suggest a similar pattern for individuals with newly diagnosed TB, including in the setting of smoked drug use. Our study was underpowered to perform multivariable assessments of factors associated with linkage to HIV or TB care, but our exploratory findings merit additional investigation in PWUD who may face increased risk of medical comorbidities and symptoms.

Our study has limitations, including small sample size that precluded multivariate analysis, reliance upon medical records rather than direct participant follow-up for LTC status, and potential lack of generalisability to other locations and populations. Furthermore, our study team actively facilitated linkage to TB care, while linkage to HIV care was passive due to differences in structures at the referring clinics. While this approach may have differentially affected the initial linkage step in the TB and HIV care cascades, this difference mirrors real-world clinical care in this setting and would not have affected subsequent care cascade steps. Further research is needed to assess LTC rates without study-provided linkage support. Finally, we did not measure distance to clinic as a potential contributor to LTC, and further investigation is needed to characterise the role of transportation barriers among PWUD in this setting.

## CONCLUSION

LTC for PWUD with HIV and TB is essential for treatment initiation and positive outcomes. We successfully linked PWUD diagnosed in the community – many of whom were asymptomatic – to TB and HIV care, though sustained engagement in care, particularly for PWH, remains challenging in this setting. As ACF increasingly identifies asymptomatic individuals, understanding their motivations and LTC barriers will be key to improving retention and outcomes.

## Supplementary Material




