# Supplementary material for: Linkage to TB and HIV care for persons who smoke illicit drugs: a prospective cohort study
Source: IJTLD Open. 2026 Feb 11;3(2):91–6. doi: 10.5588/ijtldopen.25.0515 (PMC12991689; doi:10.5588/ijtldopen.25.0515)

Supplementary Table 1: Sociodemographic characteristics by linkage to HIV care[1]

	Linked to care (N=41)	Did not link to care (N=40)	p-value
**Newly diagnosed with HIV**	17 (41.5%)	22 (55.0%)	0.223
**Age, years**	35 (29, 40)	34 (30, 40)	0.977
**Born Male**	21 (52.5%)	21 (52.5%)	0.917
**Mixed Ancestry**	33 (82.5%)	35 (87.5%)	0.562
**Education < 9th grade**	28 (68.3%)	28 (70.0%)	0.868
**Unemployed**	40 (97.6%)	37 (92.5%)	0.293
**High depression risk (CES-D)**	24 (58.5%)	26 (65.0%)	0.550
**Moderate/severe hunger (HHS)**	22 (53.7%)	19 (47.5%)	0.579
**History of Incarceration**	28 (68.3%)	20 (50.0%)	0.094
**Methamphetamine Use**	39 (95.1%)	40 (100.0%)	0.157
**Methaqualone Use**	38 (92.7%)	37 (92.5%)	0.975
**Alcohol Risk Category**			0.079
Abstains/Low	32 (78.0%)	24 (60.0%)	
Moderate/Severe	9 (22.0%)	16 (40.0%)	
**Methamphetamine/Methaqualone Risk Category**			0.484
Abstains	0 (0.0%)	0 (0.0%)	
Low	1 (2.4%)	0 (0.0%)	
Moderate	15 (36.6%)	18 (45.0%)	
Severe	25 (61.0%)	22 (55.0%)	
**Methamphetamine Risk Category**			0.320
Abstains	2 (4.9%)	0 (0.0%)	
Low	1 (2.4%)	0 (0.0%)	
Moderate	17 (41.5%)	21 (52.5%)	
Severe	21 (51.2%)	19 (47.5%)	
**Methaqualone Risk Category**			0.447
Abstains	4 (9.8%)	7 (17.5%)	
Low	3 (7.3%)	3 (7.5%)	
Moderate	15 (36.6%)	18 (45.0%)	
Severe	19 (46.3%)	12 (30.0%)	
**BMI (N=41, N=39)**			0.024
Severely Underweight/Underweight	10 (24.4%)	19 (48.7%)	
Normal Weight/Overweight	31 (75.6%)	20 (51.3%)	
**Xpert Ultra**			0.074
MTB Detected	7 (17.1%)	1 (2.5%)	
Trace MTB Detected	3 (7.3%)	2 (5.0%)	
MTB Not Detected	31 (75.6%)	37 (92.5%)	
**MTB Positive**	7 (17.5%)	0 (0.0%)	0.006
**Smear Positive**	1 (2.4%)	0 (0.0%)	0.320
**Previous TB**	17 (41.5%)	14 (35.0%)	0.550

[1]Chi-square and t-tests examined associations between variables and linkage to care group. Abbreviations: BMI - Body Mass Index; CES-D - Center for Epidemiologic Studies Depression Scale; HHS - Household Hunger Scale; MTB - *Mycobacterium tuberculosis*

Supplementary Table 2: Sociodemographic characteristics by linkage to tuberculosis care[1]

	Linked to care (N=50)	Did not link to care (N=14)	p-value
**Age, years**	37 (30, 42)	32 (30, 36)	0.063
**Born Male**	40 (80.0%)	10 (71.4%)	0.493
**Mixed Ancestry**	44 (88.0%)	13 (92.9%)	0.607
**Education < 9th grade**	29 (58.0%)	8 (57.1%)	0.954
**Unemployed**	44 (88.0%)	14 (100.0%)	0.173
**High depression risk (CES-D)**	30 (60.0%)	11 (78.6%)	0.201
**Moderate/severe hunger (HHS)**	23 (46.0%)	7 (50.0%)	0.791
**History of incarceration**	44 (88.0%)	12 (85.7%)	0.819
**Methamphetamine Use**	44 (88.0%)	14 (100.0%)	0.173
**Methaqualone Use**	47 (94.0%)	14 (100.0%)	0.348
**Alcohol Risk Category**			0.025
Abstains/Low	36 (72.0%)	14 (100.0%)	
Moderate/Severe	14 (28.0%)	0 (0.0%)	
**Methaqualone Risk Category**			0.611
Low	1 (2.0%)	0 (0.0%)	
Moderate	20 (40.0%)	4 (28.6%)	
Severe	29 (58.0%)	10 (71.4%)	
**Methamphetamine Risk Category**			0.320
Abstains	4 (8.0%)	0 (0.0%)	
Low	4 (8.0%)	0 (0.0%)	
Moderate	21 (42.0%)	5 (35.7%)	
Severe	21 (42.0%)	9 (64.3%)	
**Methaqualone Risk Category**			0.556
Abstains	4 (8.0%)	2 (14.3%)	
Low	4 (8.0%)	0 (0.0%)	
Moderate	21 (42.0%)	4 (28.6%)	
Severe	21 (42.0%)	8 (57.1%)	
**BMI (N=50, N=13)**			0.323
Severely Underweight/Underweight	23 (46.0%)	4 (30.8%)	
Normal Weight/Overweight	27 (54.0%)	9 (69.2%)	
**MTB Culture Positive**	41 (82.0%)	10 (71.4%)	0.385
**Smear Positive**	13 (26.0%)	2 (14.3%)	0.360
**Xpert Ultra**			0.218
MTB Detected	40 (80.0%)	8 (57.1%)	

Trace MTB Detected	5 (10.0%)	3 (21.4%)	
MTB Not Detected	5 (10.0%)	3 (21.4%)	
**TTP, days (N=40, N=10)**	10 (7, 16)	10 (9, 12)	0.780
**Living with HIV**	15 (30.0%)	2 (14.3%)	0.239
**Newly diagnosed with HIV (N=17)**	4 (26.7%)	1 (50.0%)	0.496
**Referral for HIV treatment**	10 (20.0%)	1 (7.1%)	0.260
**Previous TB**	22 (44.0%)	3 (21.4%)	0.126
**Asymptomatic at enrollment**	26 (52.0%)	11 (78.6%)	0.075
**Symptoms**			
Cough	18 (36.0%)	1 (7.1%)	0.037
Night sweats	3 (6.0%)	1 (7.1%)	0.876
Fever	2 (4.0%)	0 (0.0%)	0.447
Unexplained weight loss	14 (28.0%)	1 (7.1%)	0.103
**Cavitation on CXR (N=47, N=13)**	29 (61.7%)	7 (53.8%)	0.609

[1]Chi-square and t-tests examined associations between variables and linkage to care group. Abbreviations: BMI - Body Mass Index; CES-D - Center for Epidemiologic Studies Depression Scale; HHS - Household Hunger Scale; MTB - *Mycobacterium tuberculosis*; TTP - Time to positivity; CXR - Chest X-Ray

Supplemental Figure 1. Overlapping TB and HIV among participants

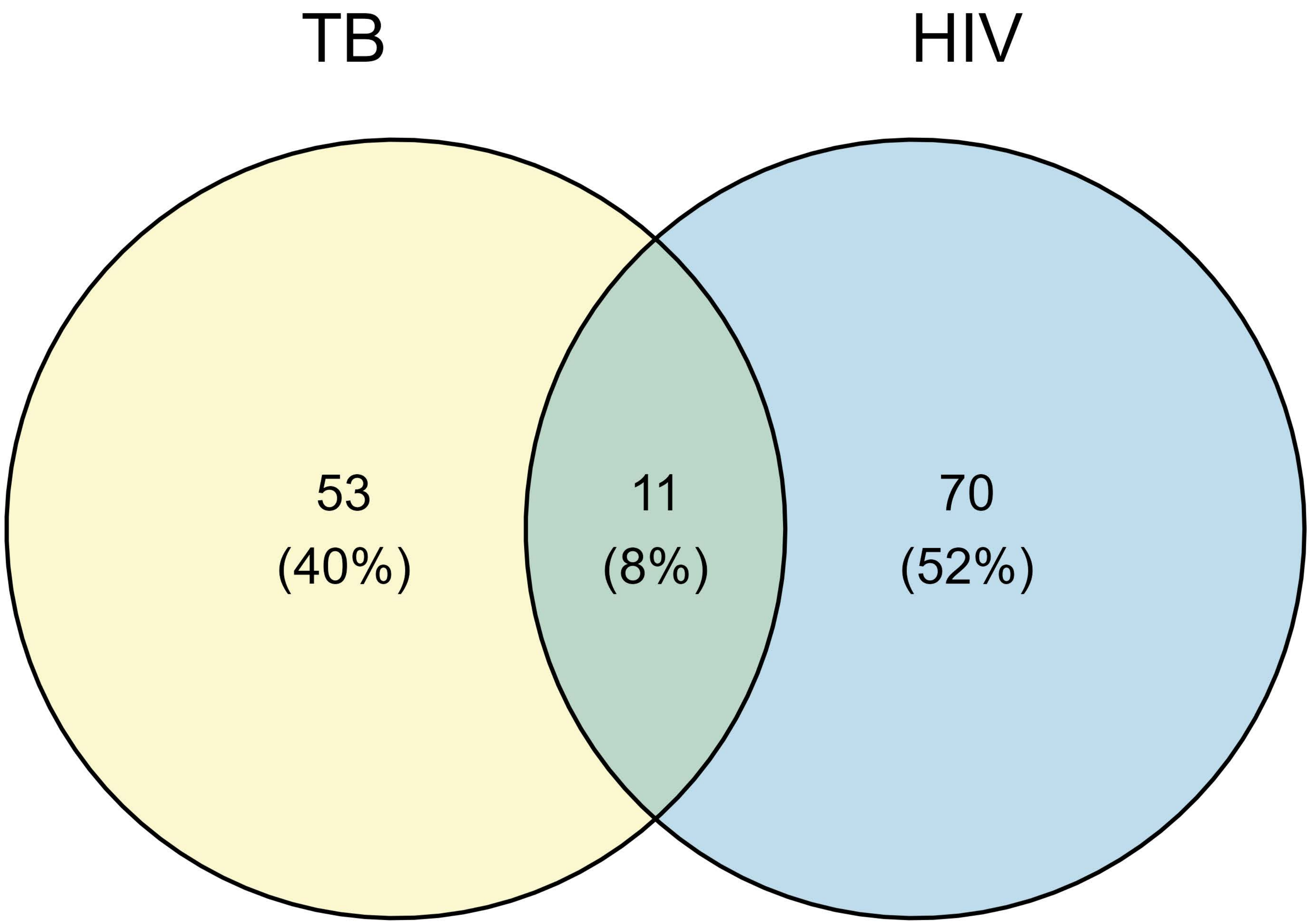

Supplement: Supplementary file 1 [file ijtldopen25-0515_supplementarydata1.pdf]
